# 非小细胞肺癌中microRNAs与EGFR-TKIs继发性耐药机制的研究进展

**DOI:** 10.3779/j.issn.1009-3419.2014.12.07

**Published:** 2014-12-20

**Authors:** 晓阳 段, 健 史

**Affiliations:** 1 050000 石家庄，河北医科大学 Graduate Student of Hebei Medical University, Shijiazhuang 050000, China; 2 050000 石家庄，河北医科大学第四医院肿瘤内科 Department of Medical Oncology, Hebei Province Cancer Hospital, Shijiazhuang 050000, China

**Keywords:** 肺肿瘤, MicroRNAs, EGFR-TKIs, 继发性耐药, Lung neoplasms, MicroRNAs, EGFR-TKIs, Secondary resistance

## Abstract

近年来，在非小细胞肺癌（non-small cell lung cancer, NSCLC）靶向治疗中，尤其是伴有表皮生长因子受体（epidermal growth factor receptor, *EGFR*）基因突变的患者，EGFR酪氨酸激酶抑制剂（tyrosine kinase inhibitor, TKI）越来越多地进入到临床治疗，但EGFR-TKI耐药的产生不仅影响药物敏感性，甚至出现疾病进展，成为制约其疗效的主要瓶颈。微小RNA（microRNAs, miRNAs）是一种非编码蛋白的RNA，参与转录后水平基因的表达调控，最近研究发现，miRNAs参与了EGFR-TKIs耐药，影响肿瘤细胞对吉非替尼的敏感性。本文就NSCLC中miRNAs与EGFR-TKIs继发性耐药之间的相关性研究进展做简要的综述。

## Abstract (en)

In recent years, in non-small cell lung cancer (NSCLC) targeted therapy, especially in patients with epidermal growth factor receptor (*EGFR*) mutations, EGFR-tyrosine kinase inhibitors (TKI) more and more come into the clinical treatment, but EGFR-TKI resistance not only influence the drug sensitivity, appear even disease progression, become the main bottleneck of its curative effect. MicroRNAs (miRNAs) is a non coding RNA and protein involved in regulating gene expression in the transcription level. Recent studies found that miRNAs involved in EGFR-TKIs resistance, which affect the sensitivity of tumor cells to treatment. In this paper, we reviewed briefly advance in miRNAs and EGFR-TKIs secondary resistance research in NSCLC.

近几年，肺癌在发达及发展中国家的发病率逐年增高，即使在发达国家，5年相对生存率也仅为16%^[1]^。肺癌患者中80%-85%的组织学类型为非小细胞肺癌（non-small cell lung cancer, NSCLC），其中约70%的患者就诊时已处于中晚期^[2]^，往往失去了手术根治的机会，只能采取以放、化疗等为基础的综合治疗。随着分子生物学研究的发展，靶向治疗逐渐用于临床并获得了良好的疗效，其中，以吉非替尼（易瑞沙）、厄洛替尼（特罗凯）为代表的肿瘤表皮生长因子受体酪氨酸激酶抑制剂（epidermal growth factor receptor-tyrosine kinase inhibitor, EGFR-TKI）的应用最为广泛，在一段治疗时间内能够获得良好的疗效，延长患者生存期，给许多中晚期肺癌患者带来了新的希望，但最终均会产生继发性耐药，导致药物敏感性差，出现疾病进展。目前耐药仍是面临的主要棘手问题之一，寻找预测肺癌患者药物敏感性的标记或解决耐药性的方法是亟待需要解决的问题，研究发现，微小RNA（microRNAs, miRNAs）为此提供了突破口，它可以影响肿瘤细胞对吉非替尼敏感性，参与EGFR-TKI耐药。因此，进一步明确miRNAs与EGFR-TKIs继发性耐药之间的关系有很重大的临床意义，不仅在预测药物敏感性，提供个体化治疗方面，甚至在解决耐药问题等方面均提供了可能。

## 1 NSCLC与EGFR-TKIs继发性耐药机制

近年来，在NSCLC分子生物学研究发展中，EGFR是目前NSCLC临床治疗研究的重要靶点之一。EGFR-TKIs在NSCLC临床治疗中，尤其是对于相对敏感的患者，无论是在一线或多线治疗均可延长患者的无疾病进展生存时间及整体生存期，并且毒副反应较轻，但是对于部分患者却无明显的疗效，而且起初对EGFR-TKIs敏感的患者，在使用EGFR-TKIs获得良好疗效一段时间（约1年-1.5年）后，均会无一例外地出现疾病进展，即所谓的继发性耐药^[3]^。

EGFR-TKIs继发性耐药涉及多种作用机制，已经明确的两种耐药机制是：①细胞内*EGFR*基因受体结合区突变（T790M），即*EGFR*基因外显子20的第790位密码子位点的氨基酸由苏氨酸（threonine, T）突变为蛋氨酸（methionine, M）。这种突变使EGFR的细胞内区结构区域发生改变，增强了催化区域与ATP的结合能力，抑制了其与EGFR-TKIs的结合，导致下游增殖相关信号不能得到有效抑制，由此产生了继发性耐药^[4]^。②*Met*基因扩增。原癌基因*Met*是编码肝细胞生长因子的受体蛋白，属于受体酪氨酸激酶家族，具有酪氨酸激酶活性。Engelman等^[5]^初次发现，在EGFR-TKIs耐药的肺癌样本中，*Met*基因出现扩增的频率较高，*Met*基因扩增占到22%。*Met*基因的扩增可不依赖于EGFR，而是通过ERBB3激活下游特定的EGFR/ERBB家族受体PI3K/AKT和RAS/ERK等信号通路，导致肺癌细胞对EGFR-TKIs产生耐药。

然而，仍有30%的NSCLC患者EGFR-TKIs耐药机制不能明确。这些耐药机制包括：①上皮间质转化（epithelial-mesenchymal transition, EMT）。上皮细胞通过特定的程序转化为具有间质表型细胞的生物学过程，导致恶性肿瘤细胞的侵袭和转移。在EMT过程中，E-钙粘蛋白和伽马连环蛋白等上皮连接蛋白的缺失，间充质波形蛋白、纤粘连蛋白等间质蛋白增多。E-钙粘蛋白主要是激活EGFR并调控下游信号通路，增强TKI药物敏感性。研究^[6]^发现，较上皮表型成分，间质表型更易发生EGFR-TKI耐药，可能是由于间质细胞引起非EGFR依赖的PI3K/AKT通路的激活，降低了肿瘤细胞增殖对EGFR的需求，从而导致了NSCLC对EGFR-TKIs产生耐药。②第10号染色体磷酸酶和张力蛋白同源缺失基因（phosphatase and tensin homolog deleted onchromosome ten, *PTEN*）基因突变。*PTEN*作为抑癌基因，其蛋白产物可以水解磷酯酰肌醇3磷酸第3位磷酸基团，抑制EGFR下游PI3K/AKT信号通路的激活，介导肿瘤细胞凋亡，发挥抑癌基因的作用。然而PTEN通过等位基因缺失、突变和甲基化等突变，导致其抑癌作用缺失。研究^[7]^发现，*PTEN*基因缺失使Erk通路异常激活，抵抗肿瘤细胞凋亡，导致EGFR-TKIs继发性耐药。③胰岛素样生长因子1受体（insulin-like growth factor 1 receptor, IGF-1R）的激活。IGF-1R是非EGFR依赖的TKI受体。由于IGF1R激活PI3K/AKT信号通路抑制TKIs药物的作用，导致了NSCLC细胞对TKI产生耐药^[8]^。但当IGF-1R信号通路受到抑制时，则可能会延缓或阻止NSCLC对TKI耐药的产生。最近Hurbin等^[9]^研究发现，在对EGFR-TKIs治疗反应上，肺粘液腺癌及非粘液腺癌类型之间是不同的。粘液腺癌过表达IGF1R（*P* < 0.000, 1）及有频繁的*KRAS*突变的倾向双调蛋白（*P*=0.004）的表达。而非粘液腺癌则过表达EGFR（*P* < 0.000, 1）和有频繁的*EGFR*突变（*P*=0.037）倾向TTF-1（*P* < 0.000, 1）的表达。高IGF1R（*P*=0.02）表达及低TTF-1（*P*=0.02）表达与吉非替尼治疗下疾病进展有关。我们发现在吉非替尼耐药H358粘液细胞中存在EGFR和IGF1R信号通路，通过抗双调蛋白及IGF1R治疗可增强H358细胞对吉非替尼诱导的细胞凋亡敏感性，从而克服粘液腺癌对EGFR-TKIs耐药。因此，在非*EGFR*突变的肺癌细胞H358中，IGF-1R同样也可引起TKI耐药的产生^[10]^。④*EGFR*扩增7号染色体的缺失。最近Furugaki等^[11]^研究发现一种新的耐药机制，即*EGFR*扩增7号染色体的缺失，通过建立对厄洛替尼耐药NSCLC细胞株进一步检测其耐药机制。研究发现，在76.47%耐药细胞中，EGFR复制数量比母细胞减少约12.5%。并且，在B10这个耐药细胞群中，几乎100%细胞为*EGFR*不扩增。约97.5%母细胞显示*EGFR*扩增，但仍有2.5%为EGFR不扩增细胞。与耐药细胞株B10相比，一种从厄洛替尼耐药细胞中孤立出来的EGFR不扩增克隆4D8也产生耐药。并且在4D8和B10中均发现了*EGFR*扩增7号染色体的损失。因此我们考虑*EGFR*扩增7号染色体的缺失为*EGFR*突变细胞株HCC827对低剂量水平厄洛替尼耐药的原因，但高剂量则会阻止耐药的发生。⑤*CRKL*基因的扩增。Suda等^[12]^研究发现，在1/11的*EGFR*突变肺癌患者的TKIs继发性耐药中发现*CRKL*基因的扩增。通过对11例TKIs继发性耐药临床样本及来自7例解剖患者的39肿瘤标本的*CRKL*基因的拷贝数量分析，发现*CRKL*基因的拷贝数量在EGFR-TKI敏感母细胞及继发性耐药的子代细胞是相同的；并且，阻止*CRKL*基因的扩增并不能逆转临床肿瘤标本EGFR-TKI耐药的发生。因此，*CRKL*基因扩增是*EGFR*突变肺癌患者的TKIs继发性耐药中罕见的耐药机制。

## 2 微小RNA（microRNAs, miRNAs）在NSCLC中的作用

miRNAs是一类由内源基因编码的长度约22个-26个核苷酸的非编码小分子RNA，参与转录后水平基因的表达调控。近年来，miRNAs成为肿瘤学研究热点，miRNAs仅占人类基因的1%，却调控了约30%的人类编码蛋白的基因，参与了包括肺癌在内的许多肿瘤的发生和发展^[13, 14]^，起到癌基因或抑癌基因的作用，另外，miRNAs在临床肿瘤诊疗过程中也具有多种用途，可以作为肺癌早期诊断的标志物、判断预后的指标，甚至还可能成为一种新的治疗手段。其异常表达、突变或异常加工均会影响靶基因miRNAs的正常功能，导致蛋白水平表达异常，继而通过影响相关的细胞信号通路改变肿瘤细胞对药物的敏感性，最近研究^[15]^发现，miRNAs参与了多种肿瘤耐药相关，尤其是NSCLC，并且可以影响肿瘤细胞对吉非替尼等药物敏感性，参与EGFR-TKIs耐药。

## 3 miRNAs与EGFR-TKIs继发性耐药关系

### 3.1 miRNAs与EGFR异常表达

EGFR异常表达会影响其对EGFR-TKIs药物的敏感性。T790M突变使TKI与EGFR结合受阻，不能有效抑制其下游增殖相关信号，从而导致EGFR-TKIs继发性耐药产生。Rai等^[16]^研究发现，表达miRNA-7质粒脂质体可以通过作用于EGFR信使RNA 3' 非翻译区而克服EGFR-TKI耐药。通过在PC-9、H3255（EGFR-TKI敏感细胞株）及RPC-9、H1975（含有T790M突变的耐药细胞株）中转染表达miRNA-7质粒，发现miR-7明显抑制这四种细胞的生长，并且胰岛素样生长因子1受体（insulin receptor substrate-1, IRS-1），RAF-1及EGFR表达均受到抑制。在应用RPC-9、H1975的小鼠异种移植模型中发现，被转染表达miRNA-7质粒的细胞受到抑制，在其余肿瘤细胞中，IRS-1、RAF-1及EGFR表达均也受到抑制。因此，转染表达miRNA-7质粒脂质体可以克服（含有T790突变）肺癌细胞中EGFR-TKI耐药情况。另外，Webster等^[8]^研究发现，miRNA-7通过与人类*EGFR*基因的3'-UTR3个位点结合，抑制*EGFR*基因的表达并能降低EGFR信号传导过程中两个关键受体AKT和（或）ERK1/2的磷酸化水平，使肿瘤细胞被阻滞于G_1_期，从而阻止细胞周期正常循环，减少细胞增殖，降低细胞活力，导致细胞凋亡。此外，Hu等^[17]^研究表明，miRNA-128在NSCLC组织及细胞中起到明显的下调作用，并且与癌的病理阶段和淋巴结转移有关，异常过度表达miR-128明显抑制肺癌细胞体外增殖、集落形成、转移及入侵，并且诱导G_1_受阻和凋亡。更重要的是，异常过度表达miR-128还可以抑制血管内皮生长因子（vascular endothelial growth factor, VEGF）-C的表达及减少包括VEGF-C 3' -非翻译区域荧光素酶的活动。NSCLC细胞过度表达miR-128及人类脐静脉内皮细胞（human umbilical vein endothelial cells, HUVECs）会导致VEGF-A、VEGFR-2及VEGFR-3（它们是肿瘤血管和淋巴血管生成关键因素）表达下降，使细胞外调控信号传导激酶（extracellular-signal regulated kinase, ERK）的磷酸化减少，抑制磷脂酰肌醇3-蛋白激酶（phosphatidylinositol-3 kinase, PI3K）/AKT和p38信号通路（其中ERK、AKT均EGFR信号传导过程中两个关键受体）。因此，miR-128在NSCLC肿瘤生成、转移、耐药等方面有重要作用，抑制miR-128不仅可以抑制肿瘤转移，而且使患者对吉非替尼等靶向药物更加敏感，并且能获得较长的生存期。

### 3.2 miRNAs与*Met*基因的扩增

在NSCLC临床治疗中，*Met*基因通过调节miRNAs的表达，参与对EGFR-TKI的继发性耐药。Garofalo等^[18]^通过对TKI耐药的*EGFR*野生型肺癌细胞株Calu-1的研究，发现*EGF*和*Met*基因均可调控miRNA-30b、miRNA-30c、miRNA-221和miRNA-222的表达，同时沉默*EGF*和*Met*基因后，4种miRNAs的表达量均明显下降，而miRNA-103和miRNA-203只受*Met*基因的调控，沉默*Met*基因后，二者的表达水平均明显上升。受*EGF*和*Met*基因双重调控的4种基因作用于TKI敏感相关的凋亡蛋白酶激活因子1（apoptotic peptidase activating factor 1, APAF-1）和细胞凋亡辅助蛋白BCL2-like 11；而miRNA-103和miRNA-203作用于TKI耐药相关的SRC[v-src sarcoma (Schmidt-Ruppin A-2) viral oncogene homolog (avian)]和蛋白激酶Cε（protein kinase Cε, PKCε），因此上述基因产物均可通过抑制或激活Met依赖的AKT和ERK信号通路影响TKI的敏感性。研究发现，*EGFR*突变型肺癌细胞株HCC827起初对吉非替尼敏感，在长期暴露于药物而产生继发性耐药后，出现*Met*基因扩增以及miRNA-30b、miRNA-30c、miRNA-221和miRNA-222的高表达及miRNA-103和miRNA-203的低表达，因此4种miRNA过表达及后2种miRNA低表达均可使肺癌细胞对TKI产生耐药。另外研究发现，使用HCC827细胞[*EGFR*基因19外显子缺少的肺腺癌细胞株]，在此细胞的基础上培养吉非替尼耐药细胞株（HCC827/GR）。检测耐药细胞株中Met的表达，并使用RT-PCR的方法检测miR-34a的表达；发现在耐药HCC827/GR细胞株中，miR-34a低表达，Met高表达，而在HCC827细胞株中，miR-34a高表达，Met低表达。TargetScan生物信息学软件提示，miR-34a可能存在655个下游靶点，Met为其中之一，*Met*基因的3′UTR的51-57及165-2171为miR-34a的结合靶点。因此，miR-34a可能通过调控靶基因*Met*而参与EGFR-TKI的继发性耐药。此外，Donev等^[19]^研究发现，抑制EGFR及MET下游分子PI3K可以通过抑制Akt磷酸化、诱导凋亡克服在*EGFR*突变肺癌细胞PC-9和HCC827由HGF介导的EGFR-TKI耐药。

### 3.3 miRNAs与EMT

EMT发生过程中，上皮表型标志物E-钙粘着蛋白（E-cadherin）表达下调，间质表型标志物波形蛋白（vimentin）表达上升，使上皮细胞失去细胞极性及与基膜连接的上皮表型，获得与细胞迁移和侵袭、抗细胞凋亡以及降解细胞外基质等能力相关的间质表型，导致上皮细胞来源的恶性肿瘤细胞获得迁移和侵袭能力，甚至参与耐药等重要生物学过程。近年来研究发现，miRNAs在EMT过程中充当关键调节者。Kitamura等^[20]^探讨EMT相关miRNAs表达及EGFR-TKI耐药之间相关性，发现TGF-β1可诱导miR-134、miR-487b及miR-655（为EMT肺腺癌细胞中位于染色体14q32的同一集落）过表达。MAGI2（membrane-associated guanylate kinase inverted 2）是这些miRNAs的靶标，同时也是PTEN的一个支架蛋白，在TGF-β1激活引起EMT的A549细胞中减少。过度表达miR-134及miR-487b可促进EMT现象发生及对吉非替尼耐药的发生。反之，则会阻止EMT现象发生及反转吉非替尼耐药。根据MiR-134/487b/655通过直接调控MAGI2表达，促进由TGF-β1诱导的EMT现象发生及影响吉非替尼耐药的发生。因此，miR-134/miR-487b/miR-655集落群在EMT现象发生的肺腺癌患者中可能成为一种新的治疗目标。另外Ahmad等^[21]^研究发现，并非所有的NSCLC患者均能从TKI药物中获益，抑制siRNA调控或者阻断Hh药理作用均能解除厄洛替尼对NSCLC的耐药性，这也导致了TGF-β1诱导的A549（A549M）增敏及间质表型H1299细胞在厄洛替尼联合顺铂治疗中所伴的肿瘤干细胞（cancer stem cell, CSC）标记物（Sox2, Nanog, EpCAM）的上调及miR-200和let-7家族miRNAs的下调，异常上调miRNAs，尤其是miR-200和let-7家族能明显减少A549M细胞对厄洛替尼的耐药。EMT细胞中由GDC-0449（是在Hh通路上的一种小分子G蛋白偶联受体的拮抗剂）介导的Hh信号抑制导致CSC标记物的衰减及miR-200和let-7家族miRNAs的上调，从而导致了EMT细胞对药物治疗的敏感性。因此，在NSCLC中，Hh信号通路通过诱导EMT导致TKI药物耐药，因此，针对Hh通路可能导致EMT表型逆转，进而增强NSCLC患者TKI药物治疗的有效性。

### 3.4 miRNAs与*PTEN*基因缺失

研究发现，*PTEN*基因缺失可能参与EGFR-TKI继发性耐药的产生。Li等^[22]^发现，将慢病毒载体转染到EGFR-TKI敏感肺腺癌PC9细胞株及耐药的PC9R细胞株中，调控miR-21的表达，通过检测由其干扰调控的PTEN和PDCD4的表达。发现miR-21在EGFR-TKI耐药细胞株PC9R中过表达。miR-21水平与PTEN和PDCD4表达相反，却与PI3K/Akt通路呈正相关。用慢病毒载体阻止miR-21可引起PC9R的凋亡，因此，我们发现miR-21通过下调PTEN和PDCD4的表达及激活PI3K/Akt通路引起NSCLC中EGFR-TKIs继发性耐药。Wang等^[23]^研究发现，miR-214在HCC827/GR（吉非替尼耐药细胞株）中明显上调，并且miR-214和PENT在HCC827/GR中相反表达，下调miR-214可改变PENT和p-AKT的表达及恢复HCC827/GR对吉非替尼的敏感性。它通过PTEN/AKT信号通路调控HCC827对吉非替尼的继发性耐药，而且抑制miR-214可能会逆转EGFR-TKIs治疗的继发性耐药。

### 3.5 miRNAs与其他相关耐药机制

Gao等^[24]^研究发现，在恶性肿瘤中，miRNAs是不受控制的小的非编码RNA，PC9非小细胞持续暴露于吉非替尼半年，获得吉非替尼耐药模型（PC9GR）。在PC9GR细胞中，发现miR-138-5p有很大比例下调。G蛋白受体124（GPR124）是miR-138-5p的直接目标。在NSCLC细胞中，GPR124的表达因miR-138-5p在蛋白及mRNA水平受到抑制。更重要是，下调GPR124会模仿miR-138-5p对吉非替尼敏感性的效果，因此，下调miR-138-5p有助于吉非替尼耐药，但恢复miR-138-5p或阻止GPR124可能会成为潜在克服NSCLC吉非替尼耐药的治疗方法。

## 4 讨论与展望

在肺癌治疗过程中，与传统的化疗相比，EGFR-TKI治疗无疑具有疗效好、毒副反应轻的特点，但是在其获得良好疗效后均会出现耐药的现象。关于肿瘤细胞对EGFR-TKI产生耐药的原因有很多方面，任何影响药物作用或干扰信号通路传导的事件均有可能导致耐药的发生。miRNA是基因表达的调控者，通过精密、复杂的信号传导网络调控人类的生命活动。因此miRNA异常可引起肿瘤耐药相关通路中基因表达水平的改变，从而破坏原有的平衡，导致耐药。目前，miRNA在肺部肿瘤耐药方面的研究已经取得一些进展，但是仍有更多方面的内容及问题有待于进一步研究。我们相信随着miRNA与肿瘤耐药关系研究的进展，miRNA将为我们在肿瘤的预测及治疗方面开拓更为广阔的空间!
